# Taxonomic and ecophysiological characteristics of actinobacteria in soils of the dry steppe zone of the Selenga Highlands (Western Transbaikalia)

**DOI:** 10.18699/VJGB-23-49

**Published:** 2023-07

**Authors:** E.P. Nikitina, L.B. Buyantueva, E.Yu. Abidueva, C.H. Sun

**Affiliations:** Baikal Institute of Nature Management of the Siberian Branch of the Russian Academy of Sciences, Ulan-Ude, Russia Banzarov Buryat State University, Ulan-Ude, Russia; Banzarov Buryat State University, Ulan-Ude, Russia; Institute of General and Experimental Biology of the Siberian Branch of the Russian Academy of Sciences, Ulan-Ude, Russia; Institute of Medicinal Biotechnology, Chinese Academy of Medical Sciences, Beijing, China

**Keywords:** chestnut soils, the Selenga Highlands, Actinomycetota, 16S rRNA gene, ecological and trophic properties of bacteria, каштановые почвы, Селенгинское среднегорье, актинобактерии, 16S рРНК, эколого-трофические свойства бактерий

## Abstract

Arid habitats have recently attracted increasing attention in terms of biodiversity research and the discovery of new bacterial species. These habitats are among the target ecosystems suitable for isolating new strains of actinobacteria that are likely to produce new metabolites. This paper presents the results on the isolation of actinobacteria from soils of the dry steppe zone of the Selenga Highlands, the characterization of their taxonomic diversity, as well as ecological and trophic properties. The bacterial counts on ISP 4 medium ranged from 6.6 × 105 to 7.1 × 106 CFU/g. The highest bacterial counts were observed in the subsurface and middle horizons of the studied soils. 28 strains of Gram-positive bacteria represented by thin-branched mycelium, coccoid and bacilliform forms were isolated. According to the results of 16S rRNA gene analysis, the isolated strains were representatives of Streptomyces, Arthrobacter, Glycomyces, Kocuria, Microbacterium, Micromonospora, Nocardioides, Pseudarthrobacter, and Rhodococcus (Actinomycetota). One isolate that showed low 16S rRNA gene sequence similarity with previously isolated and validly described species was a new species of the genus Glycomyces. It was shown that all tested strains are mesophilic, prefer neutral or slightly alkaline conditions, have growth limits in the temperature range of 5–45 °C and pH 6–9. The optimal NaCl concentration for growth of most strains was 0–1 %. The strains under study were capable of utilizing a wide range of mono- and disaccharides and polyatomic alcohols as a carbon source. The isolated strains were capable of using both organic (proteins and amino acids) and inorganic (ammonium salts and nitrates) compounds as nitrogen sources. The examinations of extracellular enzymes showed that all isolates were capable of producing catalase and amylase; 78.6 % of the total number of isolates produced protease and lipase; 53.6 %, cellulase; and 28.6 %, urease. The data obtained expand current knowledge about the diversity of microbial communities in soils of the Selenga Highlands and also confirm the potential of searching for new actinobacteria species in these soils.

## Abstract (ru)

Засушливые местообитания привлекают все больше внимания с точки зрения исследования биоразнообразия и обнаружения новых видов бактерий. Они являются одними из целевых экосистем для выделения новых штаммов актинобактерий, которые с большой вероятностью могут продуцировать новые метаболиты. В настоящей работе представлены результаты по выделению актинобактерий из почв сухостепной зоны Селенгинского среднегорья, их таксономическому разнообразию и эколого-трофическим свойствам. Численность бактерий на крахмало-аммиачной среде колебалась от 6.6 × 105 до 7.1 × 106 КОЕ/г. Максимальные значения численности были отмечены в подповерхностных и срединных горизонтах исследуемых почв. Полу- чено 28 штаммов грамположительных бактерий, представленных тонким разветвленным мицелием, кокковидными и палочковидными формами. По результатам анализа последовательностей гена 16S рРНК выделенные культуры были отнесены к родам Streptomyces, Arthrobacter, Glycomyces, Kocuria, Microbacterium, Micromonospora, Nocardioides, Pseudarthrobacter и Rhodococcus филума Actinomycetota. Один изолят, показавший низкое сходство последовательности гена 16S рРНК с ранее выделенными и достоверно описанными видами, представлял собой новый вид рода Glycomyces. Все исследуемые штаммы мезофильны, предпочитают нейтральные или слабощелочные условия, имеют границы роста в диапазоне температур от 5 до 45 °С и значений рН от 6 до 9. Оптимальная концентрация NaCl для роста культур составляла от 0 до 1 %. Исследуемые штаммы были способны утилизировать в качестве источника углерода достаточно широкий спектр моно- и дисахаридов, многоатомных спиртов. В качестве источника азота выделенные культуры использовали как органические (белки и аминокислоты), так и неорганические (соли аммония и нитраты) соединения. Исследование наличия внеклеточных ферментов показало, что все культуры могли продуцировать каталазу и амилазу, 78.6 % от общего количества изолятов продуцировали протеазу и липазу, 53.6 % – целлюлазу, 28.6 % – уреазу. Полученные данные расширяют знания о разнообразии микробных сообществ почв Селенгинского среднегорья и подтверждают, что данные почвы представляют интерес с точки зрения поиска новых видов актинобактерий

## Introduction

Actinobacteria (Actinomycetota) are a morphologically diverse group of predominantly gram-positive bacteria widely distributed in various terrestrial and aquatic ecosystems (Ventura et al., 2007; Hazarika, Thakur, 2020). They play an important role in the organic matter cycle, especially in soils, contributing to the decomposition of natural polymers such as starch, chitin, pectin, cellulose, hemicellulose and lignocellulose (McCarthy, Williams, 1992; Manucharova et al., 2004; Wang et al., 2016; Leo et al., 2018; Bao et al., 2021). Some actinobacteria species participate in the synthesis and mineralization of humus substances (Tepper, 1981; Wu et al., 2011).

In early studies, actinobacteria (in particular, actinomycetes) were considered to be unstable to the influence of extreme environmental factors, and therefore unable to occupy certain ecological niches (Kalakuckiy, Agre, 1977; Lechevalier, 1981). Later, as bacterial cultivation methods were improved, and modern molecular research methods became available, actinobacteria resistant to one or another environmental factor became known (Zenova et al., 2009, 2016; Yaradoddi et al., 2021). A large diversity of Actinomycetota is now reported in arid habitats (Kurapova et al., 2012; Zenova et al., 2014; Mohammadipanah, Wink, 2016; Xie, Pathom-aree, 2021). Certain actinobacteria can grow in soils in dry climates due to such properties as xerophilicity, resistance to ultraviolet light, mycelial structure, and spore-forming ability (Zenova, Zvyagintsev, 2002; Zenova et al., 2014; Yaradoddi et al., 2021).

In this context, the soils of the dry steppe zone of the Selenga Highlands are interesting to study because they are formed in pronounced continental and arid climates (Nogina, 1964; Batuev et al., 2000). This area is characterized by high levels of solar radiation, low and irregular precipitation, and sharp average daily and monthly fluctuations in air temperature (Chimitdorzhieva G.D., Chimitdorzhieva E.O., 2021). These conditions may have contributed to a great taxonomic diversity of actinobacteria, which may include new species and possess unique physiological mechanisms of adaptation. However, culturable soil actinobacteria of the dry steppe soils of Transbaikalia remain relatively unstudied: there are only few publications devoted mainly to the study of actinomycetes abundance (Nimaeva, 1992; Zvyagintsev et al., 1999; Buyantueva et al., 2014).

Given the above, this work aimed to isolate culturable actinobacteria from the soils of the dry steppe zone of the Selenga Highlands, to determine their taxonomic diversity, as well as ecological and trophic characteristics

## Materials and methods

Subjects of the study. Actinobacteria strains were isolated from soil samples from the dry steppe zone of the Selenga Highlands. Chestnut soils are typical of these areas, which are characterized by a sharply continental climate, long seasonal permafrost, limited rainfall (180–250 mm/year), and dry steppe vegetation. These areas are characterized by a significant accumulated temperature during the growth period (1700–1800 °С) and the length of the frost-free period (106–116 days). Winter precipitation is not more than 10 % of the annual amount, resulting in poor snow cover and prolonged spring droughts. In July and August, up to 60–70 % of the total annual precipitation falls (Nogina, 1964; Ecological Atlas…, 2015).

Four soil profiles were established. Soil profiles 1T (coordinates 51°08ʹ58.62ʺ N, 107°24ʹ25.38ʺ E; 613 m a.s.l.) and 3T (51°11ʹ15.24ʺ N, 107°34ʹ46.08ʺ E; 698 m a.s.l.) were established in the western part of the Tugnui Basin at the base of the southern slope of the Tsagan-Daban Range; soil profiles 4I (51°34ʹ50.94ʺ N, 107°03ʹ56.34ʺ E; 637 m a.s.l.) and 5I (51°37ʹ1.98ʺ N, 107°07ʹ42.06ʺ E; 686 m a.s.l.) – at the foot of the southwestern slope of the Khamar-Daban Range near the Ivolginskaya Depression.

Sampling. Soil samples were collected in the summer of 2017 according to genetic horizons. For physicochemical analyses, the soil samples were dried to an air-dry state. For microbiological studies, samples were collected in sterile containers, from three walls of each soil section in three replicates. After transportation in a cooling box, the samples were delivered to the laboratory within 12 hours. Soil samples were stored at 4 °C for no more than a week before the study. Immediately before inoculation, soil samples were dried to air-dry in a sterile laminar flow cabinet.

Physicochemical properties of soil. Soil pH was measured in water according to GOST 26423-85 (Soils. Methods for Determination of Specific Electric Conductivity, рН and Solid Residue of Water Extract); total organic carbon (TOC) content was measured according to Tyurin (Manual on Agrochemistry, 2001); total nitrogen (TN) – according to GOST 26107-84 (Soils. Methods for Determination of Total Nitrogen). Soil particle size distribution was determined using a laser diffraction particle size analyzer Analysette 22 MicroTec Plus (FRITSCH, Germany).

Pure strains isolation. Actinobacteria were isolated using a dilution plate technique. Samples were inoculated on inorganic salts-starch agar ISP 4 (Shirling, Gottlieb, 1966). The media was supplemented with nystatin (50 μg/mL) to limit the growth of fungi. The plates were incubated at 30 °C for 2–3 weeks. The actinobacteria isolates were preliminarily characterized by their morphological characteristics using a Zeiss AxioStar Plus light microscope (Carl Zeiss, Germany) with a magnification of 1000×. Further routine isolation and culturing of the dominant morphotypes were performed on yeast extract-malt extract agar ISP 2 (Shirling, Gottlieb, 1966).

DNA extraction, amplification, and sequencing of the 16S rRNA gene. DNA was isolated according to the method described by Zhou et al. (2010). Two universal primers 27F (5′-AGAGTTTGATCCTGGCTCAG-3′) and 1492R (5′-GGT TACCTTGTTACGACTT-3′) were used for the amplification of 16S rRNA gene fragments (DeLong, 1992). Amplification was performed in a reaction mixture of 50 μL containing 25 μL of 2× EasyTaq PCR SuperMix (TransGen Biotech, China), 1.5 μL of each primer (10 mM, Sangon Biotech, PRC), 2 μL DNA, and 20 μL deionized water in a Veriti™ 96‑Well Thermal Cycler DNA Amplifier (Applied Biosystems, USA). The temperature-time profile of PCR was as follows: the first cycle was 95 °C × 5 min; the subsequent 35 cycles were 94 °C × 1 min, 55 °C × 1 min, and 72 °C × 2 min; the final cycle was 72 °C × 10 min. PCR products were purified and sequenced at Sangon Biotech Company (Beijing, China) using an ABI PRISM 3730xl Genetic Analyzer (Thermo Fisher Scientific).

Taxonomic and phylogenetic analysis. The 16S rRNA gene sequence similarity was analyzed using EzTaxon- e (Yoon et al., 2017) and BLAST (Camacho et al., 2009) services. Then, the sequences of closely related species were retrieved from the GenBank database using the EzBioCloud server. Multiple sequence alignment was performed using ClustalW software. The phylogenetic trees were constructed using the neighbor-joining method using MEGA 7.0 (Kumar et al., 2016), and the branching relationships were confirmed by maximum likelihood and maximum parsimony methods. The statistical reliability of the phylogenetic reconstructions was assessed using bootstrap analysis by constructing 1000 alternative trees. The obtained nucleotide sequences were deposited in GenBank with accession numbers assigned to the strains (MN314472–MN314496, MW410748, MW410749).

Ecophysiological characteristics of the isolated bacteria. The isolates were cultured at different temperatures (5 to 55 °C, at 5 °C intervals) and NaCl concentrations (0, 1, 3, 5, 6, 7, 8, 9, and 10 %) to identify optimal parameters and growth limits. The pH range (5.0–10.0, at 0.5 intervals) was set at 30 °C by adding a buffer solution system (Xu et al., 2005). The ability to consume various carbon sources was tested according to Shirling and Gottlieb (1966). The ability to grow on a medium with organic acids was tested according to Gordon et al. (1974). The presence of extracellular enzymes (amylase, catalase, lipase, protease, cellulase, and urease), as well as the ability to release hydrogen sulfide and ammonia, were tested according to Williams et al. (1983). The tests were performed in three replicates; the corresponding sterile nutrient media were used as control samples.

## Results

Physicochemical properties of soils and the total bacterial count

The contents of total organic carbon and total nitrogen were maximum in the upper horizons of the studied soils (Table 1). Down the profile, a rather sharp decrease in the content of both indicators was observed. The pH in the upper horizons was almost neutral (6.85–7.54), while a gradual alkalization was observed down the profile. The granulometric analysis of the soils showed a predominance of light loam, except for light-humic soil, which had a sandy loam composition.

**Table 1.**
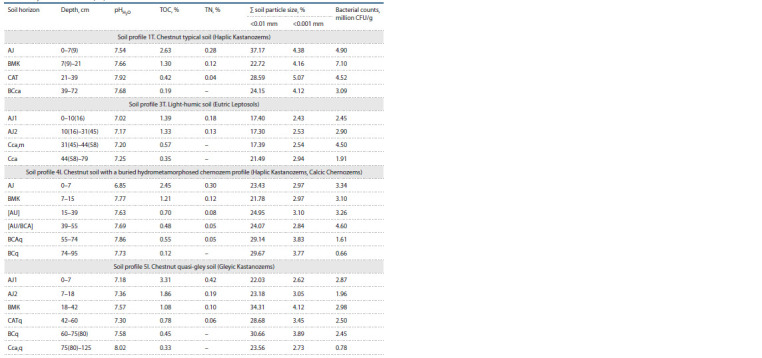
Physical and chemical properties of soils and the number of bacteria on ISP 4

The bacterial counts on ISP 4 medium reached several million colony-forming units per 1 g of soil (CFU/g soil) and ranged from 6.6×105 to 7.1×106 CFU/g. The highest bacterial counts were observed in the subsurface and middle horizons of the studied soils. Mycelial actinobacteria (actinomycetes) colonies were noticeably predominant on nutrient media, accounting for 40–80 % of the total number of bacterial colonies on the plates.

Pure strains and cell morphology of actinobacteria

34 strains of aerobic bacteria were isolated from the soil samples examined. Based on colony morphology and cell microscopy, 28 isolates were selected for further studies. The strains grown on ISP 2 medium formed rounded (0.3–1.0 cm) colonies of predominantly white, beige, yellowish, orange, brown, and maroon colors.

Several different bacterial morphotypes were observed in microscopy: cocci (4с-3-1, 5с-3-3, 13р-4-1), bacilli (3c-1-1, 6c-4-2), and branched mycelium (all other strains). Most mycelial strains were characterized by the release of water-soluble light yellow and light brown pigments into the medium.

Taxonomy and phylogeny of the isolates

Nine genera of Actinomycetota were identified as a result of 16S rRNA gene sequence analysis. Most isolates belonged to Streptomyces, a genus widely distributed in soil. Representatives of the genera Arthrobacter, Glycomyces, Kocuria, Microbacterium, Micromonospora, Nocardioides, Pseudarthrobacter, and Rhodococcus were also isolated along with them (Table 2). The isolated strains showed 98.10–100 % similarity with the previously described type strains. One isolate that showed low 16S rRNA gene sequence similarity (<98.65 %) with previously isolated and validly described species was a new species of the genus Glycomyces (Nikitina et al., 2020).

**Table 2.**
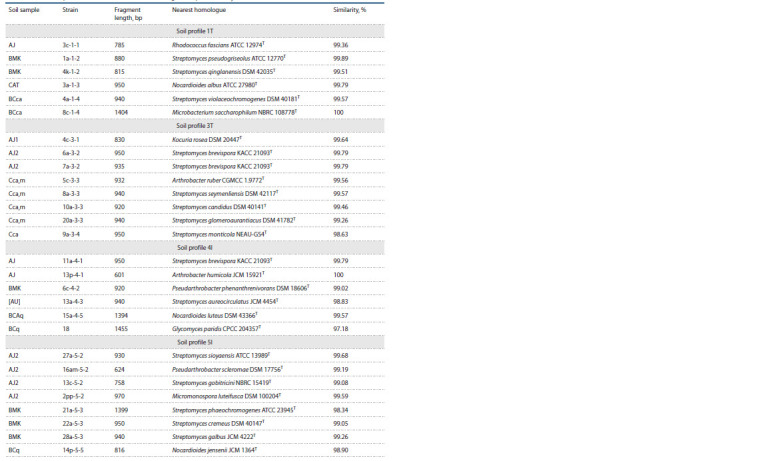
Taxonomic position of strains based on 16S rRNA gene sequences analysis

**Fig. 1.**
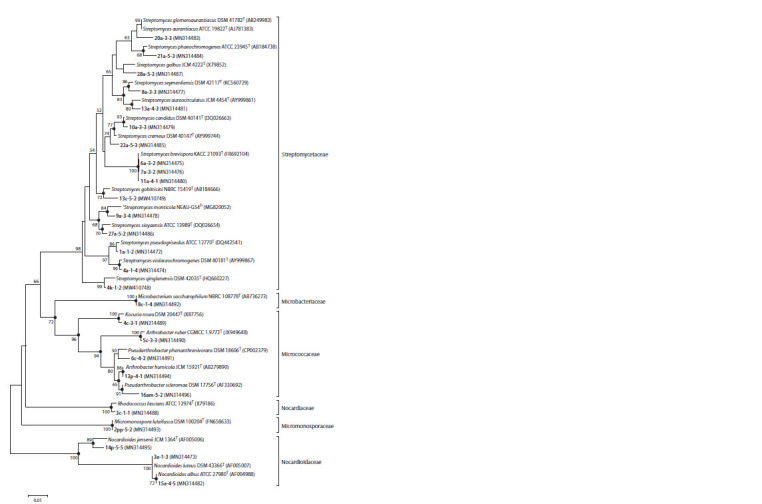
Neighbour-joining phylogenetic tree showing the phylogenetic position of actinobacteria strains Bar, 0.01 substitutions per nucleotide position. Numbers at branch nodes refer to bootstrap values based on 1000 replicates (only values >50 % are shown). Filled circles at nodes indicate corresponding branches that were recovered by using the maximum likelihood and maximum parsimony algorithms

To analyze the phylogenetic relatedness of the isolates and their closest validly described species, three phylogenetic trees were constructed using neighbor-joining, maximum likelihood, and maximum parsimony methods (Fig. 1). All three phylograms had similar basic topologies. According to the phylogenetic analysis, strains belonging to the genera Arthrobacter (5c-3-3, 13p-4-1), Kocuria (4c-3-1), Microbacterium (8c-1-4), Micromonospora (2pp-5-2) and Rhodococcus (3c- 1-1) apparently belonged to known species: it was confirmed by the high similarity (99.36–100 %) and clustering reliability (86–100 %). Strains 3a-1-3 and 15a-4-5 of the genus Nocardioides were combined with Nocardioides luteus DSM 43366T and Nocardioides albus ATCC 27980T with high confidence, respectively. Strain 14p-5-5 belonged to the subcluster uniting strains of the genus Nocardioides and demonstrated a low level of similarity with the closest homolog (98.90 %). The reliability of combining this nucleotide sequence with Nocardioides jensenii JCM 1364T into one cluster was 96 %, which implies their phylogenetic proximity. Isolates 16am-5-2 and 6c-4-2 were characterized by a high similarity with already known species. However, the low reliability of nucleotide sequence association between 6c-4-2 and Pseudarthrobacter phenanthrenivorans DSM 18606T, as well as the difference in evolutionary distance between 16am-5-2 and Pseudarthrobacter scleromae DSM 17756T do not allow a clear conclusion on the species identity of these strains.

The remaining strains, according to 16S rRNA gene sequence analysis, belonged to the genus Streptomyces and were united in one cluster with all streptomycetes collection strains on the phylogenetic tree. The 16S rRNA gene sequences of strains 6a-3-2, 7a-3-2, and 11a-4-1 were identical to each other (100 %), showing high similarity to Streptomyces brevispora KACC 21093T (99.79 %). The reliability of combining these nucleotide sequences into one cluster was 100 %, indicating that the strains could belong to this species. The same assumption may be true for strains 1a-1-2, 4a-1-4, 8a-3-3, 10a-3-3, 13а-4-3, 13с-5-2, and 4k-1-2, which had a high level of similarity with their closest homologs and clustered with them with high reliability.

Strains 27a-5-2 and 28a-5-3 showed high 16S rRNA gene sequence similarity with closely related species (99.68 and 99.26 %, respectively), but the clustering reliability was low. Strain 9a-3-4 showed a relatively low level of similarity (98.63 %) and formed a cluster with the unvalidated species Streptomyces monticola NEAU-GS4. Strain 21a-5-3 had a level of similarity with the closest described species below the threshold (98.34 %), and strains 20a-3-3 and 22a-5-3 did not form a cluster with any collection strains, despite a relatively high level of similarity with the closest homologs. These isolates could probably represent new species of the genus Streptomyces. However, the identification of streptomycetes at the species level based solely on the analysis of the 16S rRNA gene is rather complicated: an earlier study by Labeda et al. (2012) showed that the nucleotide sequences of this gene have high similarity for representatives of all taxa within the family Streptomycetaceae. Because of the complex systematics of the genus Streptomyces, which currently includes more than seven hundred validly described species, additional tests are needed to accurately determine the species identity of the isolated strains.

Ecophysiological characteristics of the actinobacteria strains

**Fig. 2.**
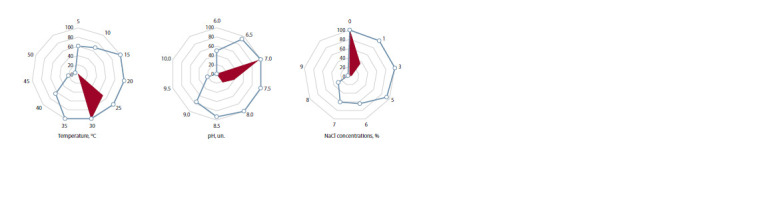
Basic growth conditions for isolated strains The values are given in % of the total number of strains. The selected sector corresponds to the optimal values of the growing conditions.

The isolated strains were characterized by different sensitivity to temperature, pH, and NaCl concentration (Fig. 2). The optimal temperature values for growth range from 25 to 30 °C, which allows us to assign the isolated strains to the group of mesophiles. In general, growth was observed in the range from 5 to 45 °C. Regarding pH tolerance, the isolates behaved predominantly as neutrophils, having growth limits from 6 to 9 with an optimum pH of 7–8. The optimal NaCl concentration for growth of most strains was 0 to 1 %. Strains 1a-1-2, 15a-4-5, 20a-3-3, 3c-1-1, 4c-3-1, 13с-5-2, 4k-1-2 and 18 were halotolerant, being able to grow at salt concentration ranging from 0 to 8 %. However, strong growth retardation of the isolated strains was observed at the NaCl concentration of 5 % or more.

Almost all strains were capable of using monosaccharides: glucose, fructose, galactose, D‑xylose, and α-rhamnose. Most were able to grow on media with disaccharides (sucrose, D‑maltose, lactose) and alcohols (glycerol, mannitol, sorbitol, dulcitol). Less than half of the strains showed the ability to use acetate and succinate. Only a few isolates were able to grow on oxalate (4a-1-4 and 6a-3-2), and citrate (6c-4-2 and 13p-4-1).

The isolated strains were capable of using both organic and inorganic nitrogen. The growth of most strains on meatpeptone broth was accompanied by the release of ammonia and hydrogen sulfide, which indicated their ability to use proteins and amino acids as nitrogen sources. The ability to assimilate ammonium salts and nitrates was detected in almost all strains, except for 4c-3-1 and 5c-3-3, which did not use ammonium salts, and 5c-3-3 with 8c-1-4, which did not use nitrates.

**Fig. 3.**
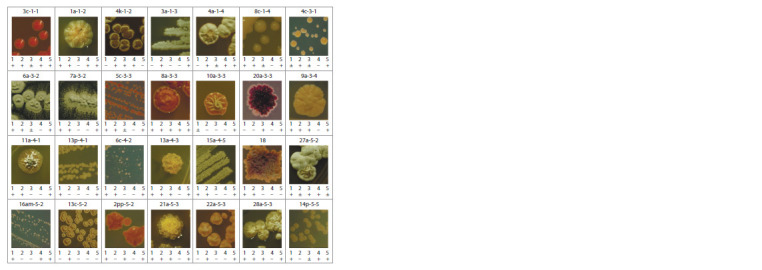
Morphology of colonies and enzymatic activity of isolated strains. 1 – protease; 2 – lipase; 3 – cellulase; 4 – urease; 5 – H2S production. (+) – positive reaction, (–) – negative, (±) – weakly positive.

All isolated strains were capable of producing catalase and amylase. The presence of protease, lipase, and cellulase was noted in most isolates. Only a few strains were able to produce urease (Fig. 3).

## Discussion

The studied soils are formed in a sharply continental climate with low precipitation and a short period of biological activity. The water regime of chestnut soils depends mainly on atmospheric precipitation and is usually unfavorable due to the light granulometric composition and gravel content in the soil. The studied soils are characterized by low stocks of total organic carbon and total nitrogen, concentrated mainly in the upper humus horizons. All this probably causes a wide distribution of oligotrophic bacteria in the microbial community, particularly mycelial prokaryotes – actinomycetes. The results obtained are consistent with earlier studies in Transbaikalia, which noted that the average actinomycete content in soils of the steppe and dry steppe zones exceeds more than half of the total abundance of cultivated prokaryotes (Nimaeva, 1992; Buyantueva et al., 2014).

As a result of this work, pure strains of actinobacteria were obtained, the closest homologs of which were isolated from soil and plant rhizosphere. Most strains were capable of forming branching mycelium. These are representatives of the genera Streptomyces, Nocardioides, Micromonospora, and Glycomyces. According to Zenova et al. (2009), such forms of actinobacteria form the basis of the hydrolytic block of prokaryotic microorganisms in soils with intermittent moisture and nutrient supply regimes. They have advantages over other bacteria, as they are capable of cell differentiation and formation of mycelium able to penetrate through phase boundaries in the soil medium.

More than half of the isolated strains belonged to the genus Streptomyces, which is quite natural: this genus is commonly associated with the soil microbiota and is most easily isolated on synthetic nutrient media. Streptomycetes strains were isolated from the upper, middle, and lower horizons of all soil sections. The widespread distribution of streptomycetes in soils is due to their mycelial structure, oligotrophy, and ability to produce arthrospores that promote dispersal and help them tolerate stress conditions (Zvyagincev et al., 2005; Cockell et al., 2013). Strains belonging to the genus Arthrobacter and the recently separated genus Pseudarthrobacter were isolated from the surface and middle horizons of the studied soils. Although their representatives do not form specific dormant forms like streptomycetes, they are capable of surviving under low-nutrient and soil desiccation conditions due to their special strategy and metabolism (Dobrovol’skaya, 2002; Wink et al., 2017). They are capable of forming cyst-like resting cells with extremely reduced metabolism under unfavorable conditions (Wink et al., 2017). One strain each of Rhodococcus, Kocuria, and Microbacterium was also isolated. Actinobacteria belonging to these genera are unable to form spores, but Rhodococcus, for example, can form mycelium capable of disintegrating into coccoid or bacilliform elements, which increase species survival (Wink et al., 2017). UV-resistant Rhodococcus (Urbano et al., 2013) and radio-resistant, psychrotrophic members of the gen

All strains under study were isolated at 30 °C, with neutral pH and negligible concentration of sodium chloride in the medium; nevertheless, they demonstrate wide limits of tolerance to these factors. This indicates their high adaptation potential to abiotic factors.

At present, the ability of actinobacteria to assimilate certain sources of carbon and nitrogen is not a significant taxonomic feature, but it can provide a basis for studying the functional role of prokaryotes in the community. The isolates exhibited broad metabolic activity to the substrates, indicating their active participation in the degradation of organic matter. Almost all isolated strains were able to consume mono- and disaccharides, and less frequently, polyatomic alcohols. The isolated strains were capable of using both organic (proteins, amino acids) and inorganic (ammonium salts, nitrates) compounds as sources of nitrogen. Amylolytic and catalytic activity was observed for all strains examined. Most isolates were characterized by proteolytic and lipolytic activity. More than half of the strains produced cellulase, and one-third produced urease.

## Conclusion

Certain characteristics of actinobacteria indicate that isolated bacteria play an important role in the degradation of organic matter and also have adaptive capabilities to environmental changes. These characteristics include features of morphology and life cycle (formation of aerial mycelium, spores, and dormant forms), the ability to use various substrates, the presence of extracellular enzymes, and a wide range of strain growth. For decades, actinobacteria have attracted the attention of many researchers due to their biotechnological potential. Further research could include additional examinations of isolated actinobacteria and evaluate the prospects of their use in biotechnology (in particular, as producers of antimicrobial components). These data not only expand knowledge about the diversity of microbial communities in soils of the Selenga Highlands but also confirm the potential of searching for new actinobacteria species in these soils.

## Conflict of interest

The authors declare no conflict of interest.
